# An All-Solid-State Nitrate Ion-Selective Electrode with Nanohybrids Composite Films for In-Situ Soil Nutrient Monitoring

**DOI:** 10.3390/s20082270

**Published:** 2020-04-16

**Authors:** Ming Chen, Miao Zhang, Xuming Wang, Qingliang Yang, Maohua Wang, Gang Liu, Lan Yao

**Affiliations:** 1 Key Laboratory on Modern Precision Agriculture System Integration Research of Ministry of Education, China Agricultural University, Beijing 100083, China; mercury@cau.edu.cn (M.C.); s20193081425@cau.edu.cn (X.W.); qingliangyang@cau.edu.cn (Q.Y.); 201023@cau.edu.cn (M.W.); pac@cau.edu.cn (G.L.); ylan2014@cau.edu.cn (L.Y.); 2 Key Lab of Agricultural Information Acquisition Technology of Ministry of Agriculture and Rural Affairs, China Agricultural University, Beijing 100083, China

**Keywords:** all-solid-state ISE, nitrate-nitrogen, AuNPs, ERGO, nanohybrids composite film, in-situ soil monitoring

## Abstract

In this paper, an all-solid-state nitrate doped polypyrrole (PPy(NO_3_^−^) ion-selective electrode (ISE) was prepared with a nanohybrid composite film of gold nanoparticles (AuNPs) and electrochemically reduced graphene oxide (ERGO). Preliminary tests on the ISE based in-situ soil nitrate–nitrogen (NO_3_^−^-N) monitoring was conducted in a laboratory 3-stage column. Comparisons were made between the NO_3_^−^-N content of in-situ soil percolate solution and laboratory-prepared extract solution. Possible influential factors of sample depth, NO_3_^−^-N content, soil texture, and moisture were varied. Field-emission scanning electron microscopy (FESEM) and X-ray powder diffraction (XRD) characterized morphology and content information of the composite film of ERGO/AuNPs. Due to the performance excellence for conductivity, stability, and hydrophobicity, the ISE with ERGO/AuNPs illustrates an acceptable detection range from 10^−1^ to 10^−5^ M. The response time was determined to be about 10 s. The lifetime was 65 days, which revealed great potential for the implementation of the ERGO/AuNPs mediated ISE for in-situ NO_3_^−^-N monitoring. In-situ NO_3_^−^-N testing results conducted by the all-solid-state ISE followed a similar trend with the standard UV-VIS method.

## 1. Introduction

Nitrogen (N) fertilizer application has greatly contributed to national food security in China in the past half-century [1]. Nitrate is the primary ionic state of nitrogen in the soil, which plays an essential role in crop protein synthesis and grain yield. Because of its water solubility, nitrate–nitrogen varies greatly with land scales and crop growth cycles and easily gets lost through runoff and leaching [2,3]. Estimations were reported that over 50% of the total reactive nitrogen load was emitted to the atmosphere, which would pose a negative impact on ecosystems and biodiversity [4,5]. Currently, the change in land use for agriculture usually causes soil erosion [6,7,8]. Additionally, for opencast agriculture, agricultural production for farmland depends on the climate environment [9,10]. For this reason, the agriculture solution under adverse climatic conditions and not cultivating under a controlled environment is the implementation of fertilizers on croplands [11,12]. Laboratory evaluation of ion-selective electrodes (ISEs) indicated the positive potential for substantial improvement for future practices. A detection precision of about 10% over a testing range of 0–100 mg/L in solution was reported. [13,14,15]. However, the ISEs were still doubted for their selectivity because of the complexity of the soil environment. The membrane should respond to the nitrate anion in the presence of various interfering ions from the soil extract. Nondestructive detections with less soil disturbance were the ideal scenarios for in-field monitoring. Commercial nitrate ion-selective electrodes (ISEs) had been equipped on the tractor or automobile trailers to achieve real-time soil NO_3_^−^-N detection; however, the correlation coefficient with the conventional laboratory spectrometry was not satisfactory for the application of site-specific fertilization. The linear correlation coefficients were reported as 0.38–0.63 [16]. Considering its service time and cost, ISE based on-the-go soil NO_3_^−^-N monitoring still faced considerable challenges.

All-solid-state ISEs eliminate the need for an internal reference electrode, resulting in benefits during miniaturization and automation [17]. Because of the electrochemical mediated imprinting and doping mechanism, nitrate doped polypyrrole (PPy(NO_3_^−^)) demonstrated improved selectivity coefficients for perchlorate, chloride, iodide and bromide anions. The nitrate doped polypyrrole (PPy(NO_3_^−^)) was one of the popular specific ion-selective membranes (ISM) used for the monitoring soil NO_3_^−^-N [18]. The detection range of PPy(NO_3_^−^) ISE was reported as 10^−1^ to 10^−5^ M with a Nernstian-slope of 54 mV/decade. The ISE based laboratory nitrate detection demonstrated close correlations with the standard spectrometric results with an accuracy of less than 10 mg/L [18]. However, the disadvantages of poor stability and lifetime primarily limited the feasibility of PPy(NO_3_^−^) ISE for in-situ detections [19]. The aqueous layer, formed between the base electrode and the ion-sensitive membrane with detections, was regarded as one of the major causes [20]. Recently, phosphonium-based ILs (Ionic-Liquids) and poly(methyl methacrylate)/poly(decyl methacrylate) (MMA-DMA) copolymer were reported for the determination of NO_3_^−^ and NH_4_^+^ in water and soil samples. The proposed ISEs exhibited a promising detection limit of 11.3 and 1.2 µm, respectively. The lifetime property of new sensors was expected to be verified for in-situ detections [21].

Graphene was employed as the mediate material to achieve conductive ion-to-electron transfer in ISE fabrication. Because of the superior hydrophobicity and stability, reduced graphene oxide (RGO) based solid-state ISEs can eliminate the occurrence of the “water layer” [22]. The problem of EMF(Electro-Motive Force) signal drift decreased to the magnitude of several μV·s^−1^. The response time performance was reported as less than 10 s. And the optimized lifetime of ISE was extended to 2–7 days [23]. Studies of gold nanoparticle (AuNP) mediated ISEs were also carried out for their similar hydrophobic capability as RGO. Enhanced biological compatibility and ion-to-electron conductivity were also observed [24]. The gold nanocluster (Au25^−^, Au25^00^, and Au25^+^) mediated solid-state K^+^ ISE even demonstrated acceptable stability performance after fabricated for one year, with proper maintenance. The characterization of surface morphology and component analysis further proved the mechanism for its stability [25]. Electrochemical biosensors, consisting of a combinate film of gold nanoparticles (AuNPs), RGO, and conductive polymer, emerged and illustrated better environmental adaptability, accuracy, and electrocatalytic performance [26]. The nanohybrids composite layer of RGO and AuNPs could be electrochemically synthesized through one-step electrodeposition [27]. Gold nanocorals and carbon nanotubes were used to make a new all-solid Li^+^ ion-selective electrode. By means of chronopotentiometry and SEM characterization, it was found that the capacitance of the conductive layer increased by 1–2 orders of magnitude, the impedance decreased significantly, the response time was less than 15 s, and the Nernst slope reached 58.70 mV/decade [28]. The shape memory polymers, which demonstrated shape recovery capability at room temperature, are worthy and challenging to be discussed for the in-situ agricultural applications [29].

Soil percolate solution, collected by porous ceramic cups, was reported as a potential useful sample for in-situ soil macronutrients monitoring, although measurement biases between the percolate solution and soil extractant were still not entirely cleared [18,30]. Our group conducted comparisons of mediate materials of electrochemically reduced graphene oxide (ERGO) and nanocarbon tubes. ISE performances of lifetime and precision were enhanced to one week and ±10% (M/M) [19,31], which illustrated overwhelming behaviors in laboratory tests. However, the current ISEs could hardly fit the requirement of robustness and stability for in-situ detection. In this study, a nanohybrid composite film of ERGO and AuNPs was fabricated to enhance the capabilities of hydrophobicity, environmental compatibility, and lifetime for in-situ ISEs based soil NO_3_^−^-N monitoring. Differences between in-situ collected soil percolate and prepared soil extractant were also analyzed (Supplementary Materials S1).

## 2. Materials and Methods

### 2.1. Reagents and Apparatus

Reagents used were all in analytical grade. Gold chloride trihydrate (HAuCl_4_·3H_2_O, ≥99.9% trace metals basis) was purchased from Aladdin (Shanghai Aladdin Bio-Chem Technology Co. Pudong, Shanghai, China). Graphene oxide (GO, 50–200 nm, 2 mg/mL) was bought from XFNANO (Jiangsu XFNANO Technology Co. Nanjing, Jiangsu, China). Pyrrole (C_4_H_5_N) was obtained from Sinopharm (China National Pharmaceutical Group Co. Haidian, Beijing, China). Potassium nitrate (KNO_3_), Sodium nitrate (NaNO_3_), phosphoric acid (H_3_PO_4_), nitric acid (HNO_3_), and PBS powders (pH = 4.0) were obtained from (Beijing Chemical Works, CO. Daxing, Beijing, China). Nitrogen gas was offered by (Beijing Hua Yuan Gas Chemical Industry, Co. Changping, Beijing, China). All of the other chemicals(Tianjin Chemical Reagents, Tianjin, China) mentioned weref rom at an analytical reagent grade. Deionized water (18.25 × 10^−6^ Ω) was applied to prepare solutions.

Electrochemical experiments were conducted using the CHI 660D electrochemical workstation (CH Instruments, Co. Shanghai, China). The reference electrode (R0303, Ag/AgCl @3.5 M KCl), counter electrode (Pt017, platinum wire, Φ1.0 mm × 37 mm), and working electrodes (GC150, Glass carbon electrode (GCE, Φ5.0 mm × 80 mm) were ordered (IDA, Tianjin, China). The pH electrode (No.9106BNWP1, Thermo Scientific Orion, MA, USA) was employed. Field-emission scanning electron microscopy (FESEM) (FE-SEM SU8020, Hitachi, Japan) was used to characterize the morphology of ISEs. X-ray powder diffraction (XRD) analysis was conducted through EPSILON5 (Malvern Panalytical, AG, Etten Leur, NEA). XRD data were analyzed with an MDI JADE 6 (Materials Data, Livermore, CA, USA).

A soil moisture sensor (ECH2O-5TE, Decagon, WA, USA) produced volumetric moisture readings. The sensor recorded with a precision of ±3% m^3^/m^3^. Standard soil chemical properties were provided by the soil testing center of the China Agricultural University with commercial analytical instruments. Detection was carried out according to the guidance of soil testing and fertilizer recommendations [32]. Soil moisture was oven-dried at the temperature of 65 °C for 12 h (SG-GDJ50, Jinghong, Shanghai, China). Soil NO_3_^−^–N was detected with a UV-VIS spectrometer (UV2450, SHIMADZU, Kyoto, Japan) at 210 nm. H_2_SO_4_ (70%) was applied to the soil extractant for acidification (Supplementary Materials S3). Soil percolate samplers (19.60.23F, Rhizon, Wageningen, The Netherlands) with the pore size of 0.58–0.65 µm were used for the collection of in-situ soil solution [33,34]. The total-N (TN) soil concentration was determined with Kjeldahl determination (KJELTEC 8400, FOSS, Hillerød, Denmark). Soil available phosphate (AP) was detected based on molybdenum blue colorimetry at 660 nm (UV2450). The organic carbon (OC) concentration was measured based on dry combustion at 550 °C for 24 h (SG-SJ1700, SIOM, Shanghai, China). Flame photometry (420, Cole-Parmer, Vernon Hills, IL, USA) was used to measure the available potassium (AK) content of the soil.

### 2.2. Preparation of the Modified Electrode

**Figure 1:**
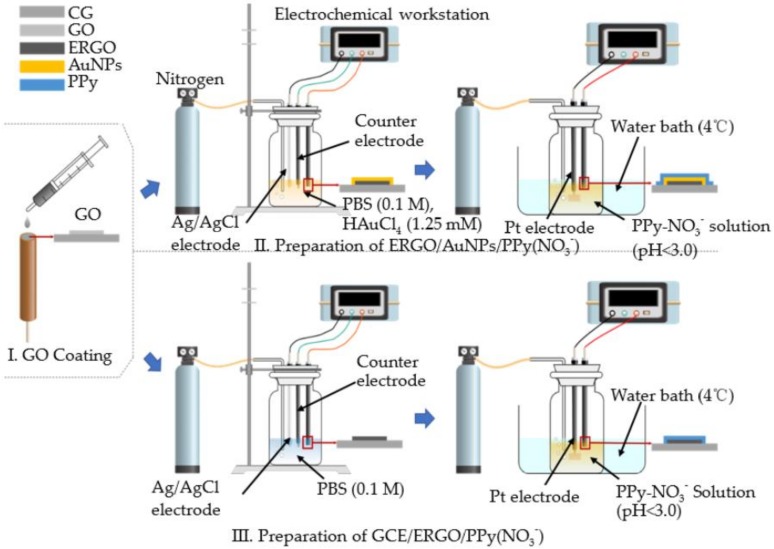
Fabrication diagram of all-solid-state ion-selective electrodes (ISEs) with mediate films of ERGO/AuNPs and electrochemically reduced graphene oxide (ERGO). Note: AuNPs are gold nanoparticles.

GCE was carefully polished with alumina slurry (particle size of 0.5 µm), sonicated with DDW (de-ionized distilled water) for 30 s, rinsed with anhydrous ethanol, and then blown with N_2_ for 1 min. All-solid-state ISEs were fabricated with two different mediate films, including a monolayer film of ERGO and a composite film of ERGO and AuNPs. The fabrication process and the membrane structure is illustrated in Figure 1.

Prepared GCE was first cast with a volume of 5 µL GO dispersion, and was blown dry. The drop-cast process was repeated two times until a uniform GO layer with a thickness of 100 µm was synthesized. Then, the mediate film deposition was conducted by the chronoamperometry method. A mixture of PBS (0.1 M, pH 4.0) and 1.25 mM HAuCl_4_ were employed as the analyte for the synthesis of ERGO and ERGO/AuNPs, respectively. A fixed potential difference of −1.4 V was applied against WE and RE for 35 min. N_2_ was purged through the analyte 10 min before the electrochemical reaction. Gently blow-dry the surface with N_2_ flow. A visible dark layer of mediate film was obtained. The ISE membrane of nitrate doped polypyrrole (PPy-NO_3_^−^) was coated onto the mediate thickness through electropolymerization by the constant current method; a current (I = 20 µA) was applied for 40 min, purging with N_2_ airflow. GCE/EGRO and GCE/EGRO/AuNPs NO_3_^−^ ISEs were tested and conditioned in 10^−2^ M NaNO_3_ solution for 12hours.

### 2.3. Soil Column and Samplers

**Figure 2:**
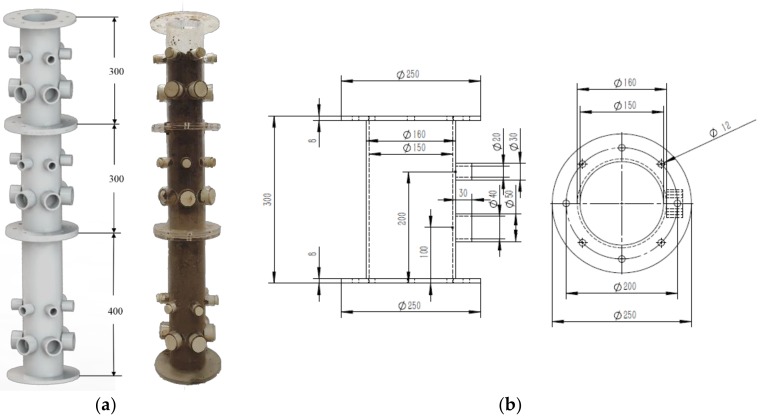
Diagram of the soil cylinder: (**a**) 3-stage design; (**b**) the drawing of the middle stage.

**Figure 3:**
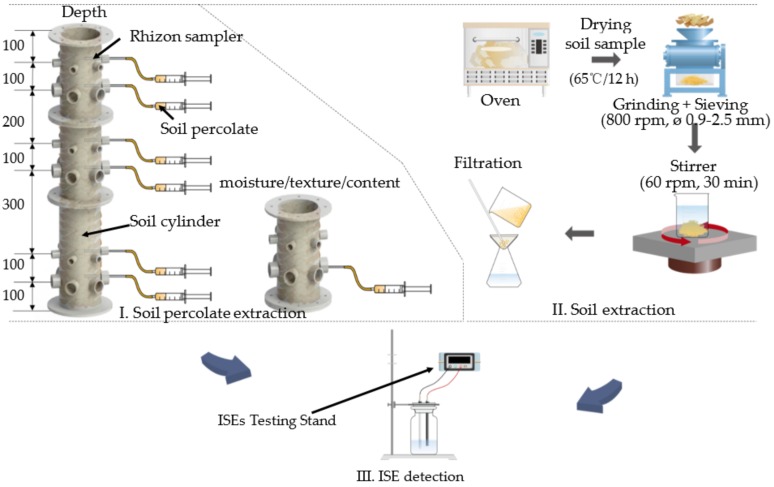
The schematic of ISE-based in-situ/laboratory soil NO_3_^−^-N detection.

A 3-stage soil cylinder was designed to simulate in-situ monitoring. The diameter of the column was 15 cm. The heights of each circular column were 30, 30, and 45 cm. The cylinder was filled with soils collected from the University extension farm, Shangzhuang, Beijing. The diagrams of soil cylinder and the schematic of how to obtain the soil solution are illustrated as Figure 2 and Figure 3. The soil cylinder was filled with uniform sandy-loam soil. The infilled soil was ideally prepared with a similar texture type to simplify the complexity of the experimental design and data analysis.

Because of the water solubility, soil NO_3_^−^-N demonstrated spatiotemporal variability. Four factors were evaluated to simulate the in-situ NO_3_^−^-N distribution, including sample depth, NO_3_^−^-N content, soil texture, and soil moisture. According to the soil cylinder design, six tested sample depths were selected. NO_3_^−^-N content variation was artificially controlled. Soil texture was controlled by pre-sieving and mixing. Two mesh servers with mesh-sizes of 0.9 and 2.5 mm were used. Moisture was manipulated by adding the pre-calculated amount of DDW to 72 h oven-dried soil with thoughtfully manually stir-mixing. Rhizon samplers were inserted into the soil cylinder. 2.5–30 mL of soil percolate solutions could be obtained after 2–6 h. Laboratory tests were carried out at the soil testing lab with soils collected close to the Rhizon sampler. Clear soil extract was processed through drying, grinding, sieving, extracting, and filtration. Soil solutions were tested with both the standard UV-VIS spectrometer and self-designed ISEs. The information about tested soil samples is summarized in Table 1.

### 2.4. ISE Performance

The cyclic voltammetries (CVs) curves of ERGO/GCE and AuNPs/ERGO were tested in potassium ferricyanide solution (0.1 M). The scanning rate was 20 mV/s and the scanning cycle was 12. Electrochemical impedance spectroscopy (EIS) was conducted in a 0.1 M NaNO_3_ solution at the scanning frequency of 1-10^−6^ Hz, AC modulation amplitude of 5 mV and the initial voltage was 0 V. The EMF readings were recorded in unstirred solutions at room temperature (25 °C) through an open circuit potentiometric test (OCPT). For testing, a 10^−5^–10^−1^ M NaNO_3_ solution was employed. ISEs were tested for a continuous 300 s for each solution. Electrochemical performances of response time, scale, and detection limit were diagnosed according to IUPAC recommendations. Stability tests were examined based on the result of potential drifts caused by the “water layer”. Experiments were carried out continuously for 2.5 h with alternate determinations in solutions with the primary anion of NO_3_^−^ and interfering anion of chloride (Cl^−^). NO_3_^−^-ISEs were tested in 10^−1^ M NaNO_3_ solution for the first 0.25 h; then detections were conducted in 10^−1^ M NaCl for another 0.5 h. Finally, another three hours of detection was continued in 10^−1^ M NaNO_3_. The stability was evaluated based on unit-time potential differences between the collected results of the first and the last tests in 10^−1^ M NaNO_3_. The Smaller variations were expected. The selectivity performance represented the preference with which the sensor responded to the analyte in the presence of various interfering ions from the sample. In this study, the matched solution method was used, as introduced by Yoshio et al. [35] and recommended by IUPAC [36]. The prime anion was fixed at 100 mL 1.0 × 10^−3^ M NaNO_3_, and the primary adding solution was 1.0 × 10^−1^ M NaNO3. The concentration of a solution with the interference anion was 1.0 × 10^−2^ mol/L. Tested ISEs were gently rinsed with DDW before use and conditioned in 10^−4^ M NaNO_3_ after detections.

ISE testing repeatability was evaluated with the recovery rate, as followed by Equation (1):

(1) $$\mathrm{Recovery}\;\mathrm{rate}=\frac{(c_{2}-c_{1})\times V_{1}}{c_{0}\times V_{0}}\times 100\%$$

where a 50 mL ($V_{1}$) of samples was used as the basic solution, then a standard solution with different concentrations ($c_{0}$) were added into the basic solution with a volume of 50 μL ($V_{0}$); $c_{1}$ represents the basic sample concentration, and $c_{2}$ represents the sample concentration after adding the standard solution.

## 3. Results and Discussions

### 3.1. Characterization of ERGO/AuNPs Nanocomposite

**Figure 4:**
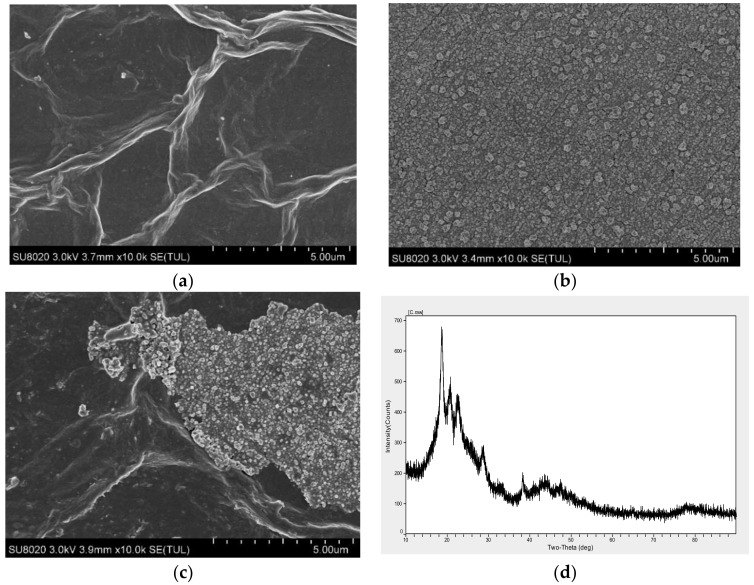
Morphology and X-ray powder diffraction (XRD) characterization of ERGO, AuNPs, and ERGO/AuNPs. (**a**) Field-emission scanning electron microscopy (FESEM) image of a layered ERGO modified on the surface of GCE in 10.0 k. (**b**) FESEM image of a layered AuNPs changed on the surface of GCE in 10.0 k. (**c**) FESEM image of ERGO/AuNPs modified on the surface of the electrode in 10.0 k. (**d**) XRD pattern of diffractogram of ERGO/AuNPs composite film.

The morphology of the surface of the electrodes modified with ERGO, AuNPs, and ERGO/AuNPs was characterized by FESEM at the same magnifications (10.0 k). As shown in Figure 4a, the ERGO film electrodeposited on the GCE was obviously a large sheet-like shape; even some ERGO nanosheets were folded together, which formed4 the typical wrinkled multilayer graphene stacks. As shown in Figure 4b, the AuNPs electrodeposited film has a rough and stereoscopic morphology on the electrode surface. The sizes of the spherical nanoparticles were 30–200 nm, with an average particle size of 115 nm. As shown in Figure 4c, a much rougher morphology was observed on the electrode surface when ERGO and AuNPs were co-electrodeposited onto the electrode. Most AuNPs were inserted into the sheet structure of ERGO, which made ERGO and AuNPs a complete nanohybrid. Hence, the contact surface area of the electrode was increased, and a conductive pathway for electron-transfer was provided. The XRD pattern of the diffractogram of GCE/ERGO/AuNPs is shown in Figure 4d. The peak appears at 2θ = 18.8°, 20.9° and 22.8°, which indexes to (0 0 4), (1 0 4) and (2 1 0) planes of carbon, respectively. The peak of 2θ = 28.9° indicates the presence of ERGO. The peak of 2θ = 38.2° is the gold. As such, the result proved that the ERGO/AuNPs nanocomposite was successfully deposited on the surface of GCE.

### 3.2. Electrochemical Performance

**Figure 5:**
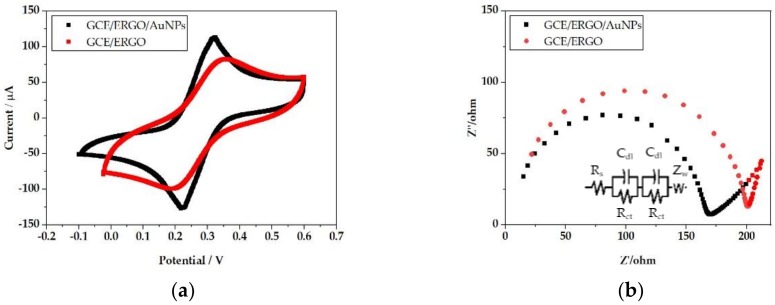

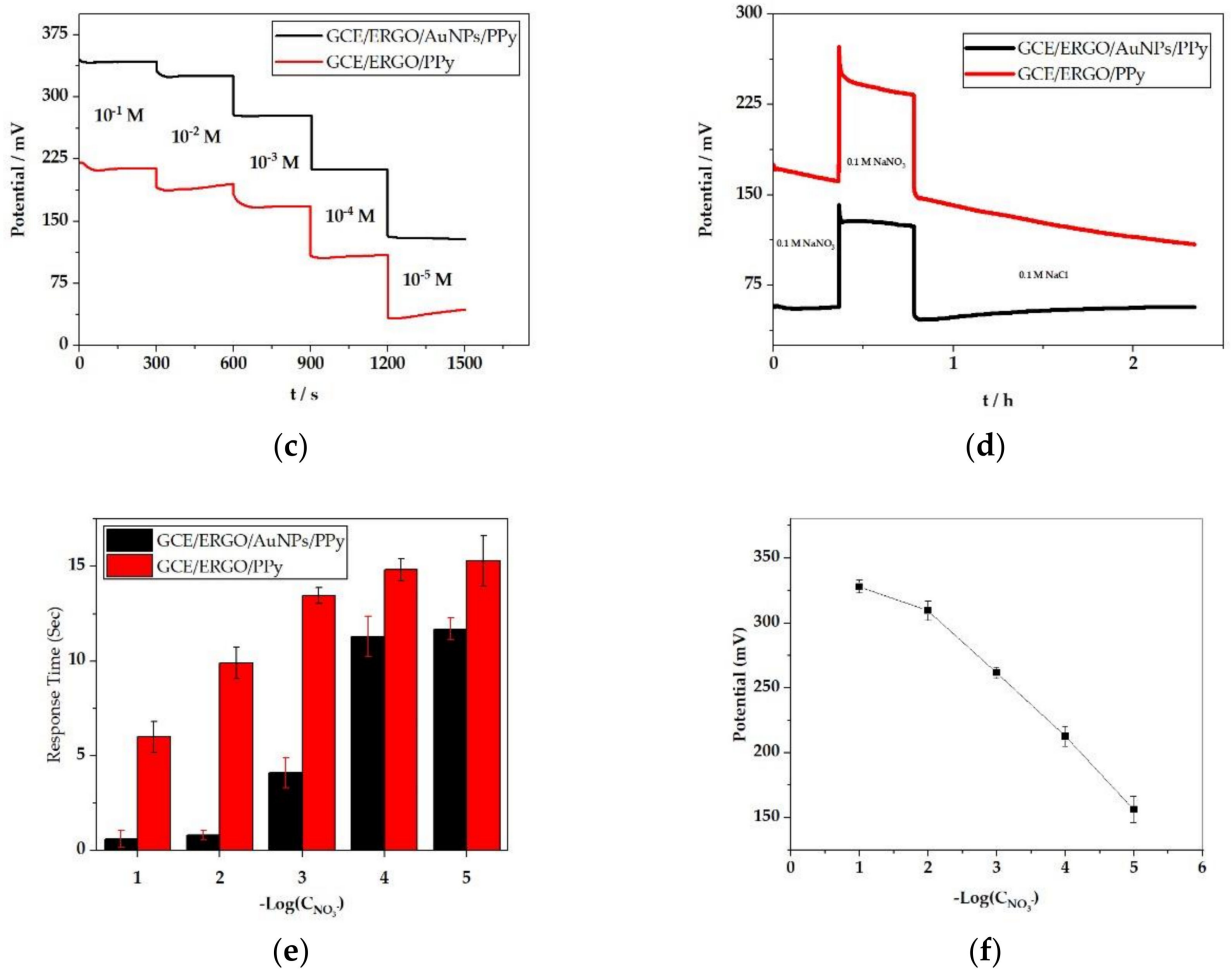
ISE Performance comparisons between two tested mediate layers of ERGO/AuNPs and ERGO (**a**) cyclic voltammetries (CVs); (**b**) EISs; (**c**) OCPT; (**d**) stability; (**e**) response time; (**f**) lifetime of the ISE with the composite mediate film (Supplementary Materials S2).

Electrochemical performances (their stability, response time, and lifetime) of CVs, EIS, and OCPT were evaluated and summarized, as shown in Figure 5. CVs curves of ERGO/AuNPs and ERGO were observed with a pair of reversible redox peaks at the scanning voltage range of −0.2–0.6 V, as shown in Figure 5a. The magnitude of redox and reversible peak currents of ERGO/AuNPs were larger than those of ERGO, which is attributed to the superior surface area property of the ERGO/AuNPs nanohybrids composite layer compared to the ERGO monolayer. The Randles model, simulated by ZsimDemo 3.30d (EChem Software, East Lansing, MI, USA), was employed to illustrate the electron transfer process on the electrode surface, as shown in Figure 5b. The equivalent circuit of the Randles model is composed of four elements, including the resistance of the electrolyte solution (R_s_), the double layer capacitance (C_dl_), the electron transfer resistance (R_ct_), and the Warburg impedance (Z_w_). The R_ct_ and C_dl_ changed 23.5% and 45.3% between ERGO/GCE and AuNPs/GCE, respectively (203.4 to 160 Ω, and 3.465 to 5.135 × 10^−9^ F). The electron transfer capability was greatly improved after employing the AuNPs into the mediate layer [37]. The evident EMF step-down decreases variated along with the increase of tested NO_3_^−^ concentration, as shown in the OCPT graph in Figure 5c. The nanohybrid composite film brought better sensitivity to the GCE/ERGO/AuNPs/PPy(NO_3_^−^) ISE with the EMF difference of 200 mV when the NO_3_^−^ concentration changed from 10^−5^ to 10^−1^ M. The EMF change in magnitude was around 160 mV. The detection limit of GCE/ERGO/AuNPs/PPy(NO_3_^−^) ISE was determined as 10^−(5.2±0.1)^ M. The composite mediate films of ERGO/AuNPs could effectively improve the sensitivity and accuracy of PPy(NO_3_^−^) ISE. Stability evaluation is shown in Figure 5d. EMF changes of the GCE/ERGO/AuNPs/PPy(NO_3_^−^) ISE between the first and the second tests of 0.1 M NaNO_3_ were calculated as 15 mV. The EMF response of GCE/ERGO/PPy(NO_3_^−^) ISE could not remain stable during the whole testing cycle. Response times of 2 ISEs are compared in Figure 5e. The composite mediate layer produced less response time. The result further proves the superior hydrophobicity of the nanocomposite mediating layer of AuNPs/ERGO, which greatly enhances the stability of ISE. The potentiometric response of tested ISEs was monitored in standard NaNO_3_ for 65 days. The GCE/ERGO/AuNPs/PPy kept its performance for the measured duration, which was an un-neglectable improvement compared to the current reported NO_3_^−^ ISEs in soil nutrient detection applications. The selectivity of the GCE/ERGO/AuNPs/PPy(NO_3_^−^) ISE is summarized in Table 2. The ISE showed the selectivity order as previously reported [35,36]. Perchlorate and halide anion brought un-neglectable disturbance on the potentiometric performance on the ISE of these sensors. The sensor illustrated satisfactory selectivity against CH_3_COO^−^, HCO_3_^−^, SO_4_^2-^ and H_2_PO_4_^−^.

### 3.3. Comparison of Soil Percolate Detection and Soil Extract Detection

**Figure 6:**
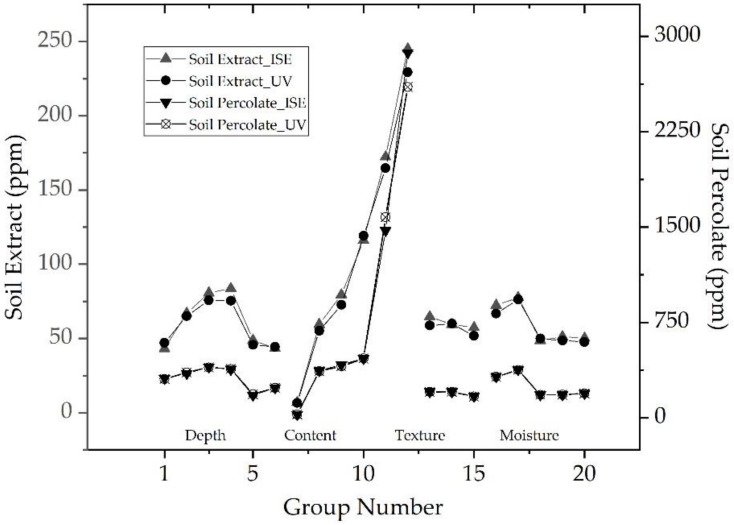
In-situ and laboratory monitoring of soil NO_3_^−^-N with the GCE/AuNPs/PPy(NO_3_^−^) ISE and UV-VIS (Supplementary Materials S2).

In-situ soil NO_3_^−^-N monitoring was conducted by making use of the GCE/AuNPs/PPy(NO_3_^−^) ISE. Variation of NO_3_^−^-N between in-situ collected soil percolate and laboratory prepared soil extracts were compared, as shown in Figure 6. Generally, testing results conducted by the all-solid-state ISE followed close correlations with the results produced by the UV-VIS method. Soil percolate contained nearly ten times high-concentrated NO_3_^−^-N than the laboratory tested “take-out” soil sample. Soil NO_3_^−^-N increased as the sample depth went deeper from 10 to 50 cm. The NO_3_^−^-N content dropped quickly at a depth of 80 and 90 cm. The GCE/AuNPs/PPy(NO_3_^−^) ISE were sensitive to the change of sample NO_3_^−^-N. Predicted NO_3_^−^-N illustrated linear growth trend with the addition of the chemical Na NO_3_. The NO_3_^−^-N content of the soil percolate obtained with the No. 12 sample (+8 u.f.) was around 2570.00 mg/kg (ppm). However, the value of the soil extract was about 230.00 mg/kg. In the test of NO_3_^—^N, the NO_3_^−^-N contents of soil percolate was increased from 170.98 to 206.50 ppm with the soil particle size reduced from over 2.5 mm to less than 0.9 mm. Laboratory soil extract kept a constant level of the NO_3_^−^-N condition with the content change from 51.75 to 59.87 ppm. Soil moisture content influences the status of water dissolved NO_3_. As illustrated in No. 16–20, soil percolate NO_3_^−^-N changed from 181.03 to 379.33 ppm. The NO_3_^−^-N content of soil extract was from 47.63 to 76.29 ppm.

The recovery rate of the ISE was tested on a randomly selected sample solution. The results are summarized in Table 3. The GCE/AuNPs/PPy(NO_3_^−^) ISE performed acceptable repeatability for soil nutrient detection, with a recovery rate of 91.2–109.7%.

## 4. Conclusions

In this study, an all-solid-state PPy(NO_3_^−^) ISE was invented with a nanohybrids composite mediate layer of ERGO/AuNPs. Electrochemical performance of the ISE was tested. The feasibility of ISE based in-situ NO_3_^−^-N monitoring was evaluated. The detection range of GCE/AuNPs/PPy(NO_3_^−^) ISE was from 10^−5^ to 10^−1^ M with the theoretical detection limit of 10^−(5.2±0.1)^ M. Compared with the monolayer of ERGO, the composite mediate films of ERGO/AuNPs could effectively improve the sensitivity, accuracy, hydrophobicity and response time of ISE. The ISE could produce stable performance with a lifetime of 65 days, which was superior to most of the all-solid-state nitrate reported ISE. The in-situ monitoring of percolate NO_3_^−^-N illustrated similar variation trends as the laboratory extracted NO_3_^−^-N in the soil cylinder test.

## Figures and Tables

**Table 1:** The soil information.

No.	Factors		Mass Moisture (%)	Nitrate Nitrogen (mg/L)	Total -N (g/Kg)	Available -P (mg/L)	Available -K (mg/L)	Organic Matter (g/Kg)
1	Depth/cm	10	19.91	61.87	6.12	5.14	12.51	10.62
2		20	20.32	68.47	6.87	5.68	11.21	10.59
3		40	21.42	75.67	7.96	4.78	16.53	10.77
4		50	21.05	70.98	7.41	4.28	15.14	10.14
5		80	19.82	47.88	4.94	5.77	13.41	10.67
6		90	20.11	52.31	6.11	6.41	15.77	10.48
7	Content/u.f. *	+0	19.17	6.55	0.90	4.43	15.01	10.65-
8		+0.5	20.54	59.44	7.12	3.42	16.11	10.47
9		+1	21.22	79.41	8.14	3.87	16.78	10.99
10		+2	19.98	116.08	13.17	3.10	15.32	11.25
11		+4	20.14	172.4	16.98	3.98	16.87	10.57
12		+8	20.54	245.16	24.13	3.52	15.64	10.42
13	Texture/mm	<0.9	21.21	51.75	5.13–5.94	7.12	16.21	10.55
14		0.9~2.5	20.41	55.47	5.19	8.79	16.22	10.55
15		>2.5	19.87	54.31	4.99	10.72	16.21	10.51
16	Moisture/g/g	10%	11.23	72.44	7.95	8.74	16.15	16.74
17		15%	15.11	77.64	7.69	8.74	16.12	16.77
18		20%	20.54	49.59	4.84	17.78	16.41	16.14
19		25%	24.67	51.20	5.23	9.14	16.74	16.62
20		30%	30.87	50.24	5.12	11.24	16.59	16.48

* u.f. Represents the unit factor. Soil moisture was controlled around the value of 20%. Standard NaNO_3_ solution was used to achieve the soil water content, where +1 u.f. was applying 0.125 g NaNO_3_ into “250 mL DDW + 1000 g pre-dried soil”. The other scale was correspondingly adjusted. The 0 u.f. group used DDW without NaNO_3_.

**Table 2:** The selectivity coefficients.

$K_{\left(NO_{3}^{-},\;\;j^{n-}\right)}\;*$	ClO_4_^−^	I^−^	Br^−^	Cl^−^	F^−^	CH_3_COO^−^	HCO_3_^−^	SO_4_^2-^	H_2_PO_4_^−^
PPy(NO_3_^−^)	1 × 10^−1^	5 × 10^−2^	1.1 × 10^−1^	3 × 10^−2^	1 × 10^−2^	5.2 × 10^−4^	5.5 × 10^−4^	5.9 × 10^−4^	6.4 × 10^−5^

* $K_{\left({\mathrm{NO}}_{3}^{-},{\;j}^{n-}\right)}$ represented for the selectivity coefficient. The primary ion is NO_3_^−^. J^n-^ is the tested interference ion, where n is the number of electric charge of J. The testing method is the matched solution method [35,36]. Note: PPy(NO_3_^−^) is nitrate doped polypyrrole.

**Table 3:** The recovery rate of GCE/AuNPs/PPy(NO_3_^−^) ISE.

Sample Info.	Detected (mg/L)	Added (mg)	Found (mg/L)	Recovery (%)
No.7-extract	6.26 ± 0.04	0.5	21.68 ± 0.39	107.91
No.9-extract	80.68 ± 2.23	5	176.49 ± 1.02	95.62
No.11-extract	172.84 ± 3.46	5	305.01 ± 1.16	105.73
No. 7-percolate	25.59 ± 0.16	2	82.52 ± 2.25	99.61
No. 5-percolate	183.72 ± 1.92	5	288.32 ± 4.02	104.6
No. 2-percolate	349.26 ± 4.28	5	471 ± 4.55	97.39

## Supplementary Materials

The following are available online at https://www.mdpi.com/1424-8220/20/8/2270/s1, S1: Graphic Abstract.jpg, S2: Testing Data.exl, S3: Soil Nitrate-Nitrogen Detection Method.docx.

## References

[1] (2019). National Stastical Yearbook. https://www.chinayearbooks.com.

[2] Lu D., Lu F., Pan J., Cui Z., Zou C., Chen X., He M., Wang Z. (2015). The effects of cultivar and nitrogen management on wheat yield and nitrogen use efficiency in the North China Plain. Field Crops Res..

[3] Li H., Liu J., Li G., Shen J., Bergström L., Zhang F. (2015). Past, present, and future use of phosphorus in Chinese agriculture and its influence on phosphorus losses. AMBIO.

[4] Hartmann T.E., Yue S., Schulz R., He X., Chen X., Zhang F., Müller T. (2015). Yield and N use efficiency of a maize-wheat cropping system as affected by different fertilizer management strategies in a farmer’s field of the North China Plain. Field Crops Res..

[5] Wang M., Ma L., Strokal M., Ma W., Liu X., Kroeze C. (2018). Hotspots for nitrogen and phosphorus losses from food production in china: A county-scale analysis. Environ. Sci. Technol..

[6] Borrelli P., Panagos P., Ballabio C., Lugato E., Weynants M., Montanarella L. (2016). Towards a pan-European assessment of land susceptibility to wind erosion. Land Degrad. Dev..

[7] Castañeda A., Alemán K., Castaño V.M. (2019). The Development of a new virtual croplands erosion measurement system using three-dimensional laser scanner and empirical Kostiakov-Lewis models. Opt. Laser Technol..

[8] Ren W., Banger K., Tao B., Yang J., Huang Y.W., Tian H.Q. (2020). Global pattern and change of cropland soil organic carbon during 1901–2010: Roles of climate, atmospheric chemistry, land use and management. Geogr. Sustain..

[9] Galdino S., Sano E.E., Andrade R.G., Grego C.R., Nogueira S.F., Bragantini C., Flosi A.H. (2015). Large-scale Modeling of Soil Erosion with RUSLE for Conservationist Planning of Degraded Cultivated Brazilian Pastures. Land Degrad. Dev..

[10] Prosdocimi M., Cerdà A., Tarolli P. (2016). Soil water erosion on Mediterranean vineyards: A review. CATENA.

[11] Seleiman M.F., Santanen A., Mäkelä P.S.A. (2020). Recycling sludge on cropland as fertilizer-Advantages and risks, Resources. Conserv. Recycl..

[12] Chen L., Redmile M., Li J., Zhang J., Xin X., Zhang C., Ma D., Zhou Y. (2019). Linking cropland ecosystem services to microbiome taxonomic composition and functional composition in a sandy loam soil with 28-year organic and inorganic fertilizer regimes. Appl. Soil Ecol..

[13] Nielsen H.J., Hansen E.H. (1976). New nitrate ion-selective electrodes based on quaternary ammonium compounds in nonporous polymer membranes. Anal. Chim. Acta.

[14] Dahnke W.C. (1971). Use of the nitrate specific ion electrode in soil testing. Soil Sci. Plant Anal..

[15] Han X.Z., Kim H.J., Moon H.C., Woo H.J., Kim Y.J. (2013). Development of a path generation and tracking algorithm for a Korean auto-guidance tillage tractor. J. Biosyst. Eng..

[16] Sibley K.J., Brewster G.R., Astatkie T., Adsett J.F., Struik P.C. (2010). In-Field Measurement of Soil Nitrate Using an Ion-Selective Electrode.

[17] Joseph V.S., Daniel F., Oliver C. (2010). Evaluation of sensing technologies for on-the-go detection of macro-nutrients in cultivated soil. Comput. Electron. Agric..

[18] Li Y., Yang Q., Chen M., Wang M., Zhang M. (2019). An ISE-based On-Site Soil Nitrate Nitrogen Detection System. Sensors.

[19] Pu P., Zhang M., Li Y., Zhang L., Ren H., Kong P., Linpei P. (2016). Preparation and Evaluation of a Stable Solid State Ion Selective Electrode of Polypyrrole/Electrochemically Reduced Graphene/Glassy Carbon Substrate for Soil Nitrate Sensing. Int. J. Electrochem. Sci..

[20] Fibbioli M., Morf W.E., Badertscher M., Rooij N.F.D., Pretsch E. (2000). Potential drifts of solid-contacted ion-selective electrodes due to zero-current ion fluxes through the sensor membrane. Electroanalysis.

[21] Choosang J., Numnuam A., Thavarungkul P., Kanatharana P., Radu T., Ullah S., Radu A. (2018). Simultaneous Detection of Ammonium and Nitrate in Environmental Samples Using on Ion-Selective Electrode and Comparison with Portable Colorimetric Assays. Sensors.

[22] Alves A.P.P., Koizumi R., Samanta A., Machado L.D., Ajayan P.M. (2016). One-step electrodeposited 3d-ternary composite of zirconia nanoparticles, RGO and polypyrrole with enhanced supercapacitor performance. Nano Energy.

[23] Umar M.F., Nasar A. (2018). Reduced graphene oxide/polypyrrole/nitrate reductase deposited glassy carbon electrode (GCE/RGO/PPy/NR): Biosensor for the detection of nitrate in wastewater. Appl. Water Sci..

[24] Wang J., Mao S., Li H.F., Lin J.M. (2018). Multi-DNAzymes functionalized on gold nanoparticles by rolling circle amplification for highly sensitive detection of thrombin on microchip. Anal. Chim. Acta.

[25] Lee C.S., Yu S., Kim T. (2018). One-step electrochemical fabrication of reduced graphene oxide/gold nanoparticles nanocomposite-modified electrode for simultaneous detection of dopamine, ascorbic acid, and uric acid. Nanomaterials.

[26] Zhang Y., Lv Z., Zhou J., Fang Y., Jiang M. (2019). Amperometric Biosensors Based on Recombinant Bacterial Laccase CotA for Hydroquinone Determination. Electroanalysis.

[27] Wang B., Ji X., Ren J., Ni R., Wang L. (2017). Enhanced electrocatalytic activity of graphene-gold nanoparticles hybrids for peroxynitrite electrochemical detection on hemin-based electrode. Bioelectrochemistry.

[28] German N., Ramanavicius A., Ramanaviciene A. (2017). Amperometric Glucose Biosensor Based on Electrochemically Deposited Gold Nanoparticles Covered by Polypyrrole. Electroanalysis.

[29] Zhang X., Zhu C., Xu B., Qin L., Wei J., Yu Y. (2019). Rapid, Localized, and Athermal Shape Memory Performance Triggered by Photoswitchable Glass Transition Temperature. ACS Appl. Mater. Interfaces.

[30] Anilanmert B., Özdemir A.A., Aydin M., Akgül M., Cengiz S. (2015). A rapid lc-ms/ms method for determination of urinary ETG and application to a cut-off limit study. Chem. Pap..

[31] Yang Q., Miao Z., Ming C., Gang L., Maohua W. (2019). All-solid-state Ca^2+^ Ion-selective Electrode with Black Phosphorus and Reduced Graphene Oxide as the Mediator Layer. Int. J. Electrochem. Sci..

[32] Liangquan W., Liang W., Zhenling C., Xinping C., Fusuo Z., Resources C.F. (2015). Basic NPK fertilizer recommendation and fertilizer formula for maize production regions in China. Acta Pedol. Sin..

[33] Falcon-Suarez I., Rammlmair D., Juncosa-Rivera R., Delgado-Martin J. (2014). Application of Rhizon SMS for the assessment of the hydrodynamic properties of unconsolidated fine grained materials. Eng. Geol..

[34] Kabala C., Karczewska A., Galka B., Cuske M., Sowiński J. (2017). Seasonal dynamics of nitrate and ammonium ion concentrations in soil solutions collected using MacroRhizon suction cups. Environ. Monit. Assess..

[35] Umezawa Y., Bühlmann P., Umezawa K., Tohda K., Amemiya S. (2002). Potentiometric selectivity coefficients of ion-selective electrodes, Part II. Inorganic Cations (Technical Report). Pure Appl. Chem..

[36] Richard P.B., Erno L. (1994). Recommendations for Nomenclature of Ion-Selective Electrodes. Pure Appl. Chem..

[37] Vinoth V., Wu J.J., Anandan S. (2016). Sensitive electrochemical determination of dopamine and uric acid using AuNPs(EDAS)–rGO nanocomposites. Anal. Methods.

