# Supplementary figures and images for: An All-Solid-State Nitrate Ion-Selective Electrode with Nanohybrids Composite Films for In-Situ Soil Nutrient Monitoring
Source: Sensors (Basel). 2020 Apr 16;20(8):2270. doi: 10.3390/s20082270 (PMC7219068; doi:10.3390/s20082270)

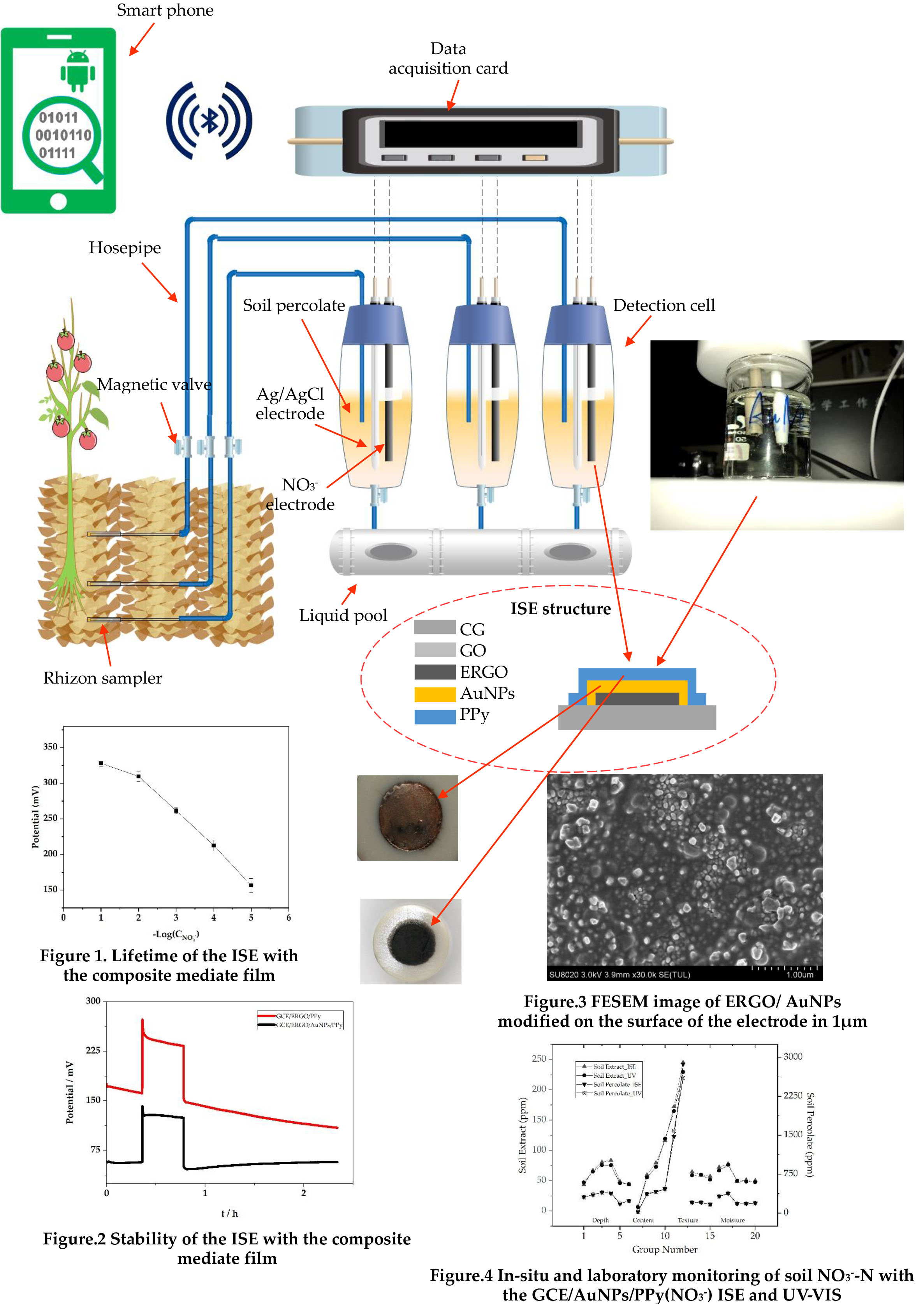

Supplement: Supplementary file 1 [file sensors-20-02270-s001.zip › Supp1-Graphic Abstract.jpg]
