# Supplementary material for: An All-Solid-State Nitrate Ion-Selective Electrode with Nanohybrids Composite Films for In-Situ Soil Nutrient Monitoring
Source: Sensors (Basel). 2020 Apr 16;20(8):2270. doi: 10.3390/s20082270 (PMC7219068; doi:10.3390/s20082270)
Supplement: Supplementary file 1 [file sensors-20-02270-s001.zip › Supp 3-Soil Nitrate-Nitrogen Detection Method.doc]

**Soil Nitrate-Nitrogen Detection**

**I UV-VIS Spectrometry**

1. **Principle**

Soil extractable NO_3_^-^-N has high absorbance at 210 nm wavelength of ultraviolet spectrophotometer. The other extractable elements, e.g. OH-, CO32-, HCO3-, and NO2-, have very low absorbance. The interference of co-existing anions can be eliminated by adding acid to neutralize acidification of leaching solution. The absorbance value of 210 nm(A210) was used for the soil extractable NO_3_^—^N detection.

The method was recommended by “National Center for Soil Measurement and Fertilization”, as discussed in Ref. 18. The soil NO_3_^-^-N results derived by UV-VIS spectrometry method was recorded as Stand_Spec_.

**2. Reagents**

2.1 Dilute the 70% H_2_SO_4_ with DDW (1：9)： 10mL of 70% H_2_SO_4_ into 90mL of DDW.

2.2 Prepare 1.00 mol·L^-1^ KCl： 74.55 g KCl into 1L DDW。

2.3 Prepare NO_3_^-^ solution [ρ(N)=100 mg·L^-1^]：0.722 g KNO_3_ into 1L DDW;

2.4 Prepare NO_3_^-^ solution [ρ(N)=10mg·L^-1^] by diluting 10.00mL of NO_3_^-^ solution [ρ(N)=100 mg·L^-1^] into 100ml with DDW.

**3. Soil Sample Preparation**

Soil sample is oven-dried at 60℃ for more than 8h until the not weight change. Weigh 10.00 g soil sample. Extract the sample with 50 mL KCl. Shaking process is conducted for 30 min. The shaking table is set to 180 rpm。Extracted soil sample solution was obtained.

**4. UV-VIS Detection**

Add 1.00 mL diluted H_2_SO_4_ into 25.00 mL target solution. Shake the mixture. Add the mixture solution into Quartz colorimetric dish. Record the A210 value.

Use the prepared NO_3_^-^ solution [ρ(N)= 10 mg·L^-1^] for the fabrication of calibration solutions with ρ(N) of 0, 1.00, 2.00, 4.00, 6.00, 8.00 mL. 1.00 mol·L^-1^ KCl is applied as extractant for dilution. Calibration model was obtained by using A210s collected at each detection.

**5、Soil NO_3_^—^N Content**

Soil NO_3_^-^-N content (mg·kg^-1^) =$\frac{\rho(N)\cdot V\cdot D}{m}$ (1)

where：Soil NO_3_^-^-N Content, mg·L^-1^；

ρ(N)-NO_3_^-^ content of the tested solution;

V-Volume of KCl, mL (50 mL in the exp.)；

D- Solution dilute ratio (D=1, if no dilution)；

m-Soil sample weight, g (10 g in the exp.).

**II Laboratory Ion-Selective Electrode Detection**

1. **Principle**

The potential difference between the Ion-Selective Electrode(ISE) and the reference electrode varies with the logarithm of molar concentration of the target ion, and could be simplified described as equation(2).

$E=K+S\log c_{i}$ (2)

where *E* is the potential difference (mV), *K* is the Nernstian intercept(mV), *S* represents the Nernstian response slope(mV decade^-1^; decade means 10 times of the concentration change; the ideal value is 59.16 mV/z for a monovalent ion at 25℃), and *c_i_* is the molar concentration (mol L^-1^).

The soil NO_3_^-^-N results derived by laboratory ISE was recorded as ISE_LT_ .

**2. Reagents**

2.1 Prepare NO_3_^-^ solution [ρ(N)=1000 mg·L^-1^]：7.221 g KNO_3_ into 1L DDW;.

2.2 Prepare NO_3_^-^ solution [ρ(N)=500/ 100/ 50/ 10/ 1 mg·L^-1^] by diluting the prepared [ρ(N)=1000 mg·L^-1^] NO_3_^-^ solution with DDW.

**3. Soil Sample Preparation**

Soil sample is oven-dried at 65℃ for more than 8-12h until the not weight change. Weigh 10.00 g soil sample. Extract the sample with 25 mL DDW. Shaking process is conducted for 15 min. The shaking table is set to 180 rpm. Clear extracted soil sample solution was obtained after filtration.

**4. ISE Detection**

Immerge the ISE into the testing solution. Record the E obtained. Calculate the NO_3_^-^ by Equation discussed in Eq.(3).

ISE_LT_ content (mg·kg^-1^) = *c_i_ × 14 × 1000/ N* (3)

Where：Soil NO_3_^-^-N Content, mg·L^-1^；

*c_i_* – solution NO_3_^-^-N content, mol·L^-1^；

N- DDW to soil ratio (2.5 in the exp.);

Calibration was conducted before testing. Build the ISE calibration model by using the prepared NO_3_^-^ solution [ρ(N)=500/ 100/ 50/ 10/ 1 mg·L^-1^.

**III In-situ Ion-Selective Electrode Detection**

1. **Principle**

ISE detection principle is the same as Eq. (2).

The in-situ soil NO_3_^-^-N results derived by ISE with soil percolate solution.

**2. Reagents**

2.1 Prepare NO_3_^-^ solution [ρ(N)=1000 mg·L^-1^]：7.221 g KNO_3_ into 1L DDW;.

2.2 Prepare NO_3_^-^ solution [ρ(N)=100/ / 10/ 1 mg·L^-1^] by diluting the prepared [ρ(N)=1000 mg·L^-1^] NO_3_^-^ solution with DDW.

**3. Soil Sample Preparation**

Percolate solutions were collected with Rhizon samplers from the 3-stage soil cylinder.

**4. ISE Detection**

Immerge the ISE into the testing percolate solution. Record the E obtained. Calculate the NO_3_^-^ by Equation discussed in Eq.(2).

Calibration was conducted before testing. Build the ISE calibration model by using the prepared NO_3_^-^ solution [ρ(N)=500/ 100/ 50/ 10/ 1 mg·L^-1^.
